# Uroguanylin increases Ca^2+^ concentration in astrocytes via guanylate cyclase C-independent signaling pathway

**DOI:** 10.3325/cmj.2021.62.250

**Published:** 2021-06

**Authors:** Nikola Habek, Martina Ratko, Aleksandra Dugandžić

**Affiliations:** 1 Laboratory of Cellular Neurophysiology, Croatian Institute for Brain Research, Zagreb University School of Medicine, Zagreb, Croatia; 2 Center of Excellence for Basic, Clinical and Translational Neuroscience, Zagreb University School of Medicine, Zagreb, Croatia; 3 Department of Physiology, Zagreb University School of Medicine, Zagreb, Croatia

## Abstract

**Aim:**

To investigate the cyclic guanosine monophosphate (cGMP)/guanylate cyclase C (GC-C) -independent signaling pathway in astrocytes, which are a suitable model due to their lack of GC-C expression.

**Methods:**

Patch clamp was performed and intracellular Ca^2+^ concentrations and pH were measured in primary astrocyte cultures and brain slices of wild type (WT) and GC-C knockout (KO) mice. The function of GC-C-independent signaling pathway in the cerebellum was determined by behavior tests in uroguanylin (UGN) KO and GC-C KO mice.

**Results:**

We showed for the first time that UGN changed intracellular Ca^2+^ levels in different brain regions of the mouse. In addition to the midbrain and hypothalamus, GC-C was expressed in the cerebral and cerebellar cortex. The presence of two signaling pathways in the cerebellum (UGN hyperpolarized Purkinje cells via GC-C and increased intracellular Ca^2+^ concentration in astrocytes) led to a different motoric function in GC-C KO and UGN KO mice, probably via different regulation of intracellular pH in astrocytes.

**Conclusion:**

The UGN effects on astrocytes via a Ca^2+^-dependent signaling pathway could be involved in the modulation of neuronal activity.

Guanylin peptides (GPs: guanylin [GN] and uroguanylin [UGN]), members of the natriuretic peptide family, are secreted after a meal in the gut lumen and blood (1-3). They activate guanylate cyclase C (GC-C), which increases the intracellular concentration of cyclic guanosine monophosphate (cGMP), followed by the activation of cGMP-dependent protein kinase G (PKG). In the intestine, GC-C is a receptor for the heat-stable enterotoxin of *Escherichia coli* (STa) (4). However, GC-C was also discovered in extra-intestinal tissues that are not exposed to STa, such as the kidneys, reproductive system, brain, and lungs (5,6), so the existence of endogenous GC-C activators was assumed. In 1992, GN was isolated from the rat intestine, and a year later UGN was isolated from opossum urine (7,8).

In the brain, natriuretic peptides play an important role in neuronal differentiation, neuromodulation, and neuroprotection, but the role of GC-C and GPs is still unknown. GC-C is expressed in midbrain dopaminergic neurons of the substantia nigra compacta and ventral tegmental area. Its activation increases the firing frequency induced by metabotropic glutamate and muscarinic acetylcholine receptors. Therefore, GC-C knockout (KO) animals develop attention deficit hyperactivity disorder (ADHD)-like behavior with increased locomotor activity and seeking behavior (9). Furthermore, GC-C is expressed in pro-opiomelanocortin (POMC)-expressing neurons of the arcuate nucleus of the hypothalamus, where it changes feeding behavior, the activity of brown adipose tissue, and energy balance (10-13).

The existence of a cGMP/GC-C-independent GP signaling pathway was suggested two decades ago. The binding sites for STa in the intestine are not completely co-localized with GC-C expression. Therefore, in the intestine there exist two types of binding sites for STa. The first is a GC-C-dependent binding site, which is a low-affinity binding site (14). The second, a GC-C-independent site, is a high-affinity binding site representing 10% of all STa-binding sites. This additional signaling pathway is present in GC-C KO mice, and its activation increases the intracellular Ca^2+^ concentrations (15). In 1993, Mann et al showed the existence of a GC-C-independent signaling pathway for STa in cultured rat small intestine epithelial cells. The authors concluded only that this novel signaling pathway was cGMP-independent, without suggesting which other signaling pathways could be involved (16). Since GPs are natriuretic peptides, it is unsurprising that UGN KO animals have increased blood pressure (17). Hypertension likely develops because GC-C-independent signaling pathway is not activated in the kidney, where this pathway has been predominantly investigated (18-21).

The Ca^2+^-dependent signaling pathways in astrocytes have many functions: they participate in tripartite synapses, regulate neuronal circuits, and affect the behavior (22). In this study, we assessed whether UGN was involved in the modulation of neuronal activity in different brain regions via a Ca^2+^-dependent signaling pathway in astrocytes. Since astrocytes are diverse and brain-region specific, we evaluated the physiological importance of the activation of cerebellar astrocyte Ca^2+^ signaling pathway by UGN in motoric function regulation.

## Methods

### Animals

The animals used were male wild-type (WT) mice of C57Bl/6 strain. GC-C KO and UGN KO mice were generated (C57Bl/6 background) as described previously (10,23). The experiments were carried out on 4-6-month-old male mice, and primary astrocyte cultures were isolated from newborn (postnatal day 0) WT animals.

We used WT and GC-C KO littermates only when necessary, to minimize animal suffering and reduce the number of experimental animals. Mice were fed with standard rodent chow and were given water and food *ad libitum*. Temperature was maintained at 23 °C and humidity between 50% and 75%, with day/night cycles of 12 h.

WT, UGN KO, and GC-C KO mice were anesthetized with intraperitoneal injections of 2, 2, 2-tribromoethanol (250 mg/kg, Sigma-Aldrich, St. Luis, MO, USA) (IACUC Guidelines: Anaesthesia) and transcardially perfused with oxygenated (95%O_2_/5%CO_2_) ice-cold N-methyl-D-glucamine (NMDG) artificial cerebrospinal fluid (aCSF) containing 93 mM NMDG, 93 mM HCl, 2.5 mM KCl, 1.2 mM NaH_2_PO_4_, 30 mM NaHCO_3_, 20 mM HEPES, 10 mM MgSO_4_, 0.5 mM CaCl_2_, and 25 mM glucose, as described previously (24). The brain was quickly isolated and sliced for electrophysiological and Ca^2+^ measurements.

### Primary astrocyte culture isolation by magnetic activated cell separation

WT mice pups (postnatal day 0) were anesthetized on ice, which is an IACUC standard procedure, and decapitated when their skin became light blue. After the brains were carefully removed and the meninges stripped off, the brains were placed in StemPro^®^ Accutase^®^ (Thermo Fisher Scientific, Waltham, MA, USA) enzyme solution for 60 min at room temperature. The enzyme reaction was stopped with the same volume of Dulbecco's Modified Eagle's Medium/F12 (DMEM/F12, Thermo Fisher Scientific). Supernatant-containing cells were collected and centrifuged for 6 min at 300 g. The supernatant solution was removed, and cell pellet was subjected to magnetic activated cell separation, as previously described (Astrocytes Isolation Starter Kit, Cat. No. 130-096-054, Miltenyi Biotec GmbH, Bergisch Gladbach, Germany) (25).

Isolated astrocytes were plated on coverslips and cultured in DMEM/F12, with an addition of 10% fetal calf serum, 100 U/mL penicillin, and 100 μg/mL streptomycin, and maintained in an atmosphere of 5% CO_2_/95% air at 37 °C. The cells were used 3-10 (6.9 ± 0.5, n = 12) days after isolation. For RNA isolation, astrocytes were re-suspended in TRI Reagent® Solution (Thermo Fisher Scientific).

### Reverse transcription polymerase chain reaction (RT-PCR)

Total RNA was isolated from primary astrocytes, brain regions, and the intestines of WT mice with TRI Reagent® Solution (Thermo Fisher Scientific). After sacrifice by cervical dislocation, the brains and intestines were isolated. The cerebral cortex, cerebellum, hypothalamus, and midbrain were carefully removed from the brain. Total RNA (1 μg) from cells or tissues was used for cDNA synthesis (GoScript Reverse Transcription System, Promega, Madison, WI, USA). PCR was performed using cDNA (1 μL) and the following primer sets: GC-C S: 5′TGCGCTGCTGGTGTTGTGG3′, AS:5′CCCGAGGCCTGTCTTTTCTGTAA3′ (product size 341 bp); GAPDH S: 5′ACGGCCGCATCTTCTTGTG3′; AS: 5′CCCATTCTCGGCCTTGACTG3′ (product size 235 bp) in the following conditions: 2 min at 94 °C, 30 s at 58.8 °C, 1 min at 72 °C (1 cycle); 30 s at 94 °C, 30 s at 58.8 °C, 1 min at 72 °C (30 cycles). The primer set for GC-C was designed to give equal product size for both GC-C isoforms. PCR products were analyzed by agarose gel electrophoresis. Glyceraldehyde 3-phosphate dehydrogenase (GAPDH) expression was used as cDNA control, and the negative control was the reaction mixture without cDNA. PCR products were verified by sequencing.

### Immunohistochemistry

WT and GC-C KO mice were anesthetized as described above and transcardially perfused with PBS and 4% paraformaldehyde (PFA). The brains were isolated and put in 4% PFA for 24 h and cryoprotected in 20% and 30% sucrose in PBS. Four-micrometer slices were cut on cryostat Leica CM3000.

After rehydration in PBS and antigen retrieval (5 min in boiling 10 mM citrate buffer, pH = 6), permeabilization in 0.2% Tween-20 in PBS for 8 min was performed. The sections were blocked for 1 h at room temperature with 1% BSA in PBS and incubated with a primary antibody against GC-C (1:25, Santa Cruz, Santa Cruz, CA, USA, sc-34428) at +4 °C over night. After three washes with PBS, the sections were incubated with a secondary antibody (1:500, Alexa fluor 488, Thermo Fisher Scientific) for 1 h at room temperature. After washing, the sections were incubated overnight with another primary antibody, anti-NeuN (1:1000, Abcam plc., Cambridge, UK, ab104225) or anti-GFAP (1:1000, DAKO, Agilent Technologies, Santa Clara, CA, USA, Z 0334) at +4 °C. After three washes in PBS for 10 min, the sections were incubated with a secondary antibody (1:200, Cy5, Jackson ImmunoResearch Laboratories, Inc., West Grove, PA, USA, code: 711-175-152) for 1 h at room temperature. After washing, they were mounted by fluorescent mounting medium (DAKO).

Fluorescent signals were acquired by Zeiss LSM 510-META (Zeiss, Oberkochen, Germany) confocal microscope. Alexa fluor 488 was excited by 488 nm argon laser line, and the fluorescent emission was collected from 505-530 nm. Cy5 labeling glial fibrillary acidic protein (GFAP) or specific neuronal marker neuronal nuclei marker (NeuN) was excited by 633 nm HeNe laser line, and the fluorescent signal was collected from 650-680 nm.

### Electrophysiology

Coverslips with astrocytes were placed in the recording chamber and perfused with HCO_3_^-^-free aCSF containing 154 mM NaCl, 1.25 mM NaH_2_PO_4_, 2 mM MgCl_2_, 3 mM KCl, 2 mM CaCl_2_, and 10 mM glucose. Patch pipettes (5-7 MΩ) were filled with an internal solution containing 115 mM K-gluconate, 20 mM KCl, 1.5 mM MgCl_2_, 10 mM phosphocreatine, 10 mM HEPES, 2 mM Mg-ATP, and 0.5 mM GTP. Freshly made nystatin (160 μM) was added to the internal solution to permeabilize the cell membrane. The starting resistance of the prepared pipettes was 5.3 ± 0.3 MΩ, n = 4, and the liquid junction potential was compensated before establishing the cell-attached mode. The cells were visualized under upright microscope Axioskop 2 FS plus (Zeiss), and the membrane potentials were recorded in perforated whole-cell configuration by SEC 0.5LX npi amplifier (npi electronic GmbH, Tamm, Germany) and WinEDR software (University of Strathclyde, Glasgow, UK).

For electrophysiological and Ca^2+^ measurements on brain slices, WT, UGN KO, and GC-C KO mice were anesthetized as described above and transcardially perfused with oxygenated (95%O_2_/5%CO_2_) ice-cold NMDG aCSF. The brain was quickly isolated and cut into 300-μm thick slices (Vibratome 1000 plus, The Vibratome Company, St. Louis, MO, USA) in ice-cold NMDG-aCSF. The initial recovery was performed in the same solution at 32 °C for 10 min followed by additional recovery for at least 60 min at room temperature in oxygenated aCSF: 128 mM NaCl, 1.25 mM NaH_2_PO_4_, 26 mM NaHCO_3_, 2 mM MgSO_4_, 3 mM KCl, 2 mM CaCl_2_, and 10 mM glucose before use.

The cerebellar slices of WT and GC-C KO mice were placed in the recording chamber and perfused (2-3 mL/min at 33 ± 1 °C) with oxygenized aCSF: 127 mM NaCl, 10 mM D-glucose, 1.25 mM NaH_2_PO_4_, 26 mM NaHCO_3_, 1 mM MgCl_2_, 3 mM KCl, and 2 mM CaCl_2_. Purkinje cells were identified under differential interference contrast (DIC) as large cells between the granular and molecular layers of the cerebellar cortex. We used the same internal solution as described before; the size of patch clamp pipette was 6.0 ± 0.6 MΩ, n = 13. After establishing a seal, the cell membrane was mechanically ruptured.

### Ca^2+^ imaging

Astrocytes were incubated with 10 μM Fluo-4 am in Hanks' Balanced Salt Solution (HBSS, Sigma-Aldrich) at 37°C in 5%CO_2_/95% air for 15 min and washed with HBSS before imaging.

Brain slices were loaded with 0.5 μM of sulforhodamine 101 (SR101 – when applied) and 10 μM of Fluo-4 am dye (26) or Oregon Green 488 BAPTA-1 am (both from Thermo Fisher Scientific) in oxygenated aCSF containing 100 mM of mannitol for 20 min and recovered for 10 min in oxygenated aCSF at room temperature. Two to three brain slices were used for each imaged region per animal.

The imaging was performed with a Zeiss LSM 510 META confocal microscope. Slices or cells on coverslips were placed in the recording chamber and excited using 488 nm argon laser line, and the fluorescent emission was collected above 520 nm. The SR101 was excited using 543 nm HeNe laser line, and the fluorescent emission was collected above 560 nm. The acquired rate was 1 Hz. Fluorescent signal intensity was analyzed with MATLAB (MathWorks, Natick, MA, USA) and presented as ΔF/F_0_. The Ca^2+^ response in astrocytes was determined by measuring only the SR101 positive cells.

### pH measurements

Astrocytes on coverslips were loaded with 10 μM BCECF, AM (Thermo Fisher Scientific) in HBSS for 15 min in 5%CO_2_/95% air at 37 °C and washed before imaging. The coverslips were put in the recording chamber and placed on inverted microscope Axiovert 10 (Zeiss). The cells were excited with two fluorescent wavelengths at 436 nm and 488 nm, and the emissions were detected at 520-560 nm with a single-photon-counting tube (H3460-04; Hamamatsu, Herrsching, Germany). The results were analyzed with Biofluor software and presented as a fluorescence ratio 488/436 nm.

**Figure 7:**
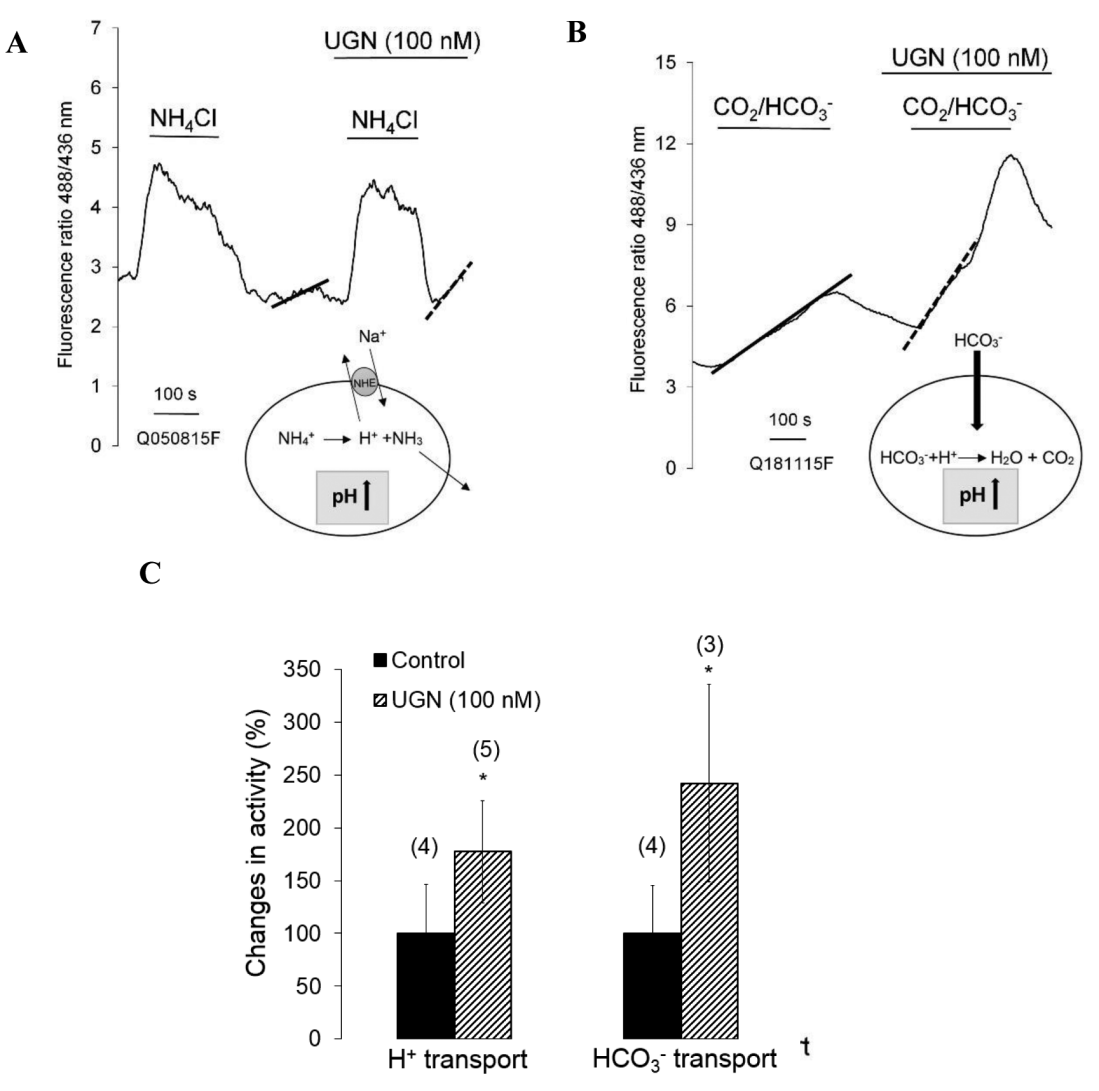
Uroguanylin (UGN, 100 nM) changes the H^+^ and HCO_3_^-^ transport in primary astrocyte cultures. UGN increases the Na^+^/H^+^ exchanger activity measured by ammonia pulse (**A**) and HCO_3_^-^ transport (**B**); summarized effects (**C**). The mechanism of cytoplasm alkalinization due to the activation of Na^+^/H^+^ exchanger and HCO_3_^-^ transport is presented. The mean of the slopes of the control experiments was set to 100%. The results are shown as mean ± standard deviation (SD), n = 3-5, primary culture isolated from three newborn animals. Bar represents 100 s. Asterisk indicates significant difference compared with controls, *P* < 0.05. Continuous line – control; dashed line – UGN.

Na^+^/H^+^ exchanger (NHE) activity was tested by ammonia pulse (27). Cells mounted on the recording chamber were perfused with HCO_3_^-^-free aCSF. After initial recording, 20 mM NH_4_Cl was added. During an ammonia pulse, NH_3_ enters the cells and binds H^+^ ions, leading to alkalization. After removal, NH_3_ cells acidify, and NHE normalizes pH values by H^+^ transport (Figure 7A). HCO_3_^-^ transport was tested after the initial cell perfusion with HCO_3_^-^-free aCSF followed by perfusion with saturated aCSF (containing 26 mM NaHCO_3_) with 5%CO_2_/95%O_2_. Cells were alkalized due to HCO_3_^-^ transport.

### Behavior tests

*Hanging wire test* was performed as previously described (28). The animals grab the middle of a 38-cm long and 2-mm thick wire fixed at the height of 49 cm. The latency until the fall was recorded. The maximum experiment duration was 30 s, and the following number of points was assigned: 1-5 s – one point; 6-10 s – two points; 11-20 s – three points; 21-30 s – four points; more than 30 s or when mice reached the end of wire – five points. If the recorded time was less than 5 s, eg, when the animals did not hold on to the wire properly, the experiment was repeated three times to get a better score. All animals that scored five points on 2-mm wire (easy) repeated the experiment on a 4-mm wire (intermediate). Again, the animals that scored five points continued the experiment on a 6-mm wire (hard). All points were added up. The maximum score was 15.

*Rota-rod test* was performed using Rota-Rod Model57604, with a 3-cm diameter rod (Ugo Basile SRL, Gemonio, Italy) (29). After a training period of 30 s at 5 rpm, the rod was accelerated over 5 min from 5 to 40 rpm. The endpoint was the time when the mouse fell from the rod or was not able to walk on it. The first experiment or test-one was followed by three trials on two consecutive days with the resting time for at least 6 h.

### Statistical analysis

For electrophysiological, Ca^2+^, and pH experiments, the animal groups consisted of a minimum of three animals. For behavior tests, six animals per group were used, as previously reported (30). The data are presented as median and interquartile range (IQR) or mean ± standard deviations (SD). Normality of distribution was tested with the Kolmogorov-Smirnov test. The *t* test was used to compare each group with its control. If more than two parameters were compared, ANOVA with a *post-hoc* Tukey test was used. The correlations were assessed with the Pearson correlation test. Data obtained by hanging wire behavior test were analyzed with the Kruskal-Walls test and a *post-hoc* Dunn test. *P* values ≤0.05 were considered significant. Statistical analysis was performed with the GraphPad Instat (GraphPad Software, San Diego, CA, USA).

## Results

### Uroguanylin increases intracellular Ca^2+^ concentration in different brain regions

Since natriuretic peptides affect Ca^2+^ signaling in the brain cells (31) via the activation of phospholipase C, we first determined if UGN increased the intracellular Ca^2+^ concentration in the brain. We performed Ca^2+^ imaging experiments on the brain of adult WT and GC-C KO littermates.

**Figure 1:**
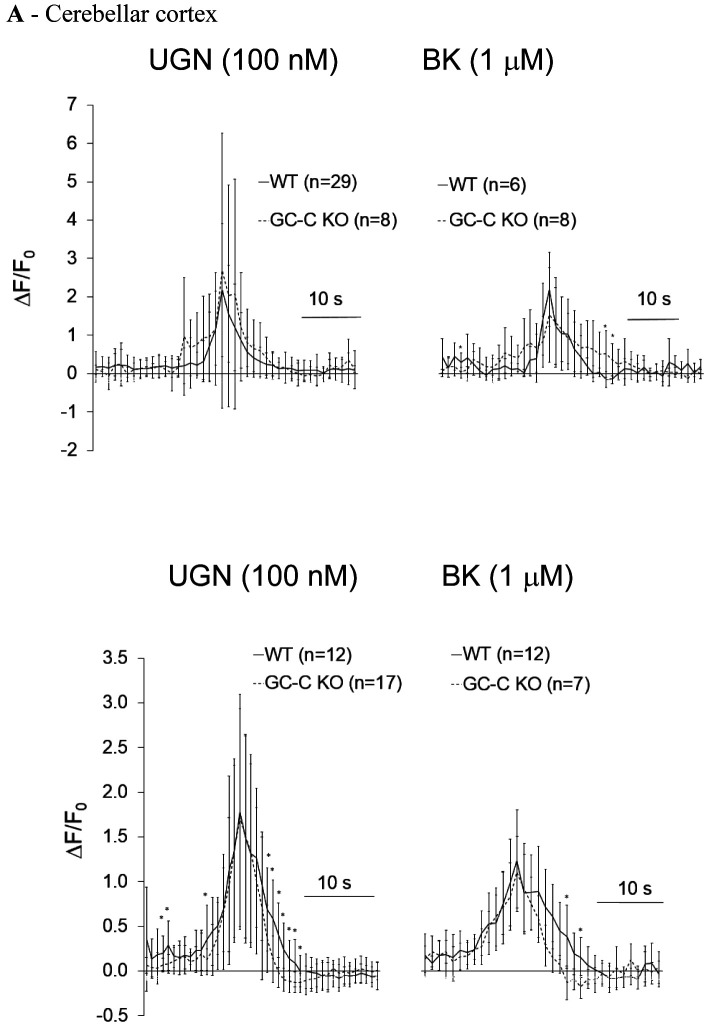
Uroguanylin (UGN) increases intracellular Ca^2+^ concentration in different brain regions. The effects of UGN (100 nM) (left) and bradykinin (BK, 1 μM) (right) were observed in the cerebellar cortex (**A**), wild type [WT] = solid line; guanylate cyclase C knockout [GC-C KO] = dashed line) and cerebral cortex (**B**). The experiments were performed in three animals per group for each brain region. The results are shown as mean ± standard deviation (SD). Asterisk indicates a significant difference between WT and GC-C KO mice, at *P* < 0.05 level. Bar represents 10 s. ΔF/F_0_ is change of light output in time (ΔF) over initial brightness (F_0_).

In the cerebellar cortex, UGN (100 nM) increased the intracellular Ca^2+^ cell concentrations in both molecular and granular layers in WT and GC-C KO animals (Figure 1A left). Bradykinin (BK, 1 μM) was used as a positive control (Figure 1A right) (32). In the cerebral cortex, UGN (Figure 1B left) and BK (Figure 1B right) increased the intracellular Ca^2+^ concentrations in both WT and GC-C KO animals. The increase in Ca^2+^ concentrations lasted significantly longer in WT compared with GC-C KO mice.

### Expression of guanylate cyclase C in the brain

**Figure 2:**
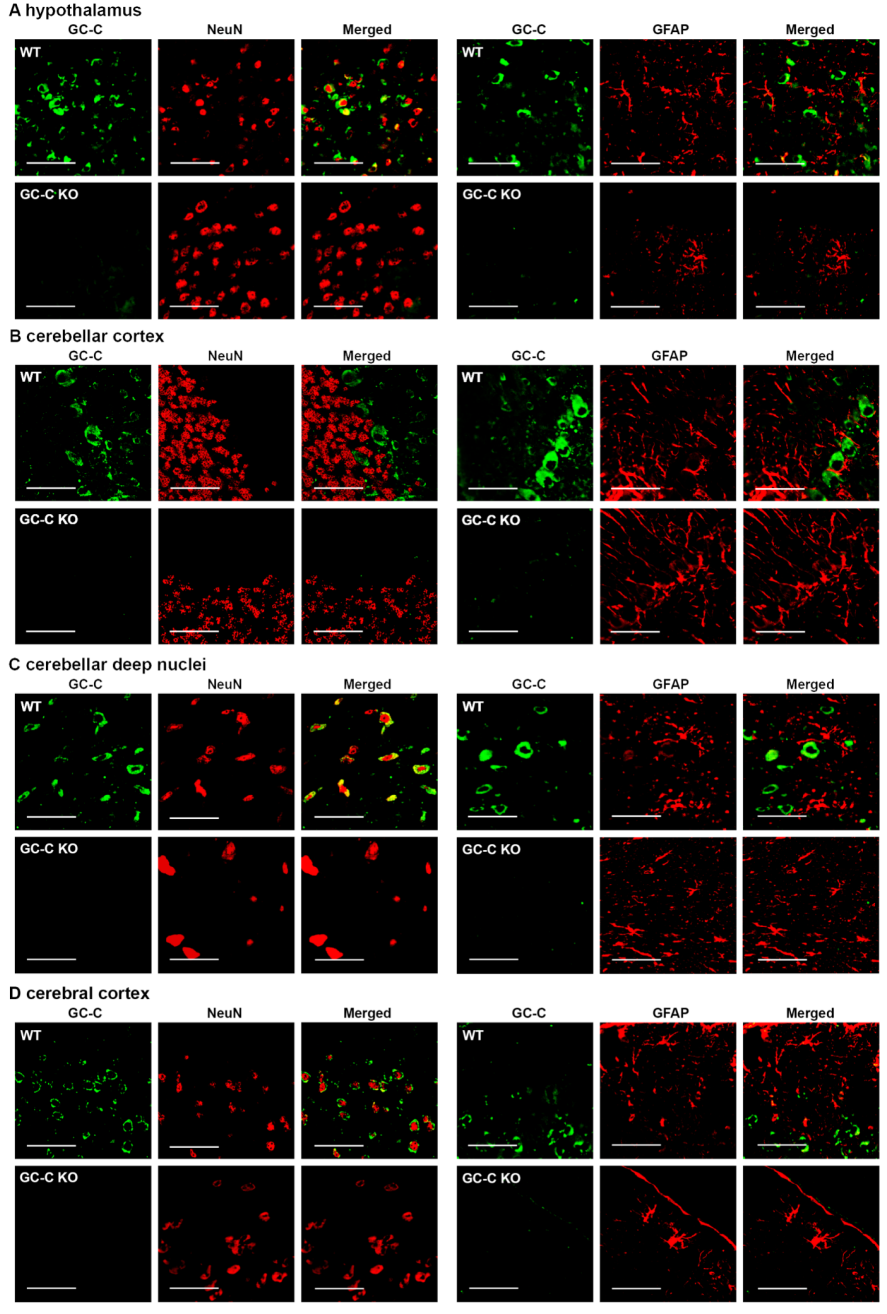
Guanylate cyclase C (GC-C) is expressed in neurons of different brain regions. GC-C (green) co-localized with a neuronal nuclei marker (NeuN = red, left panel) but not with the astrocytes marker, glial fibrillary acidic protein (GFAP = red, right panel) in (**A**) the hypothalamus, (**B**) cerebellar cortex, (**C**) cerebellar deep nuclei, and (**D**) cerebral cortex of wild-type mice. GC-C knockout (KO) mice were used as a negative control. Bar represents 50 μm.

As recently published (12), GC-C is expressed in POMC neurons of the hypothalamic arcuate nucleus. To determine which cells express GC-C in the cerebral cortex and cerebellum, where we found the Ca^2+^-dependent signaling pathway for UGN, we performed co-localization experiments with GFAP, a specific astrocyte marker, and NeuN, a specific neuronal marker. GC-C was not found in astrocytes because it was not co-localized with GFAP. GC-C was expressed on the cell membranes of hypothalamic neurons (Figure 2A), cerebellar Purkinje cells (Figure 2B), neurons of the cerebellar deep nuclei (Figure 2C), and the cerebral cortex (Figure 2D), as previously shown in the human prefrontal cortex (33). GC-C KO animals were used as negative control.

### The function of signaling pathways for uroguanylin in the cerebellum

**Figure 3:**
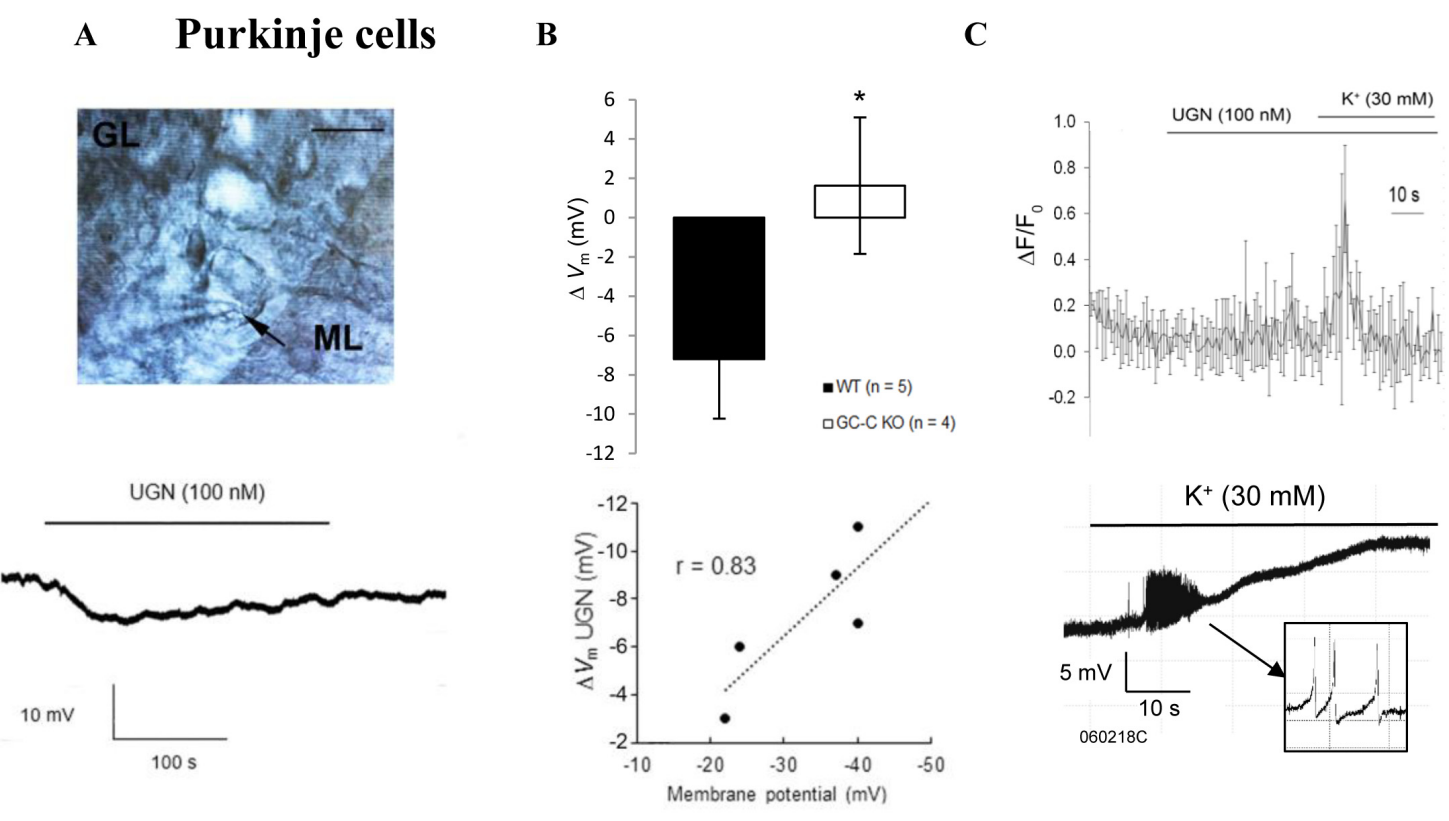
Only uroguanylin (UGN) guanylate cyclase C (GC-C)-dependent signaling pathway is present in the Purkinje cells of the cerebellar cortex. UGN (100 nM) in the cerebellum hyperpolarized Purkinje cells (**A**) – upper panel represents a differential interference contrast, DIC, scan of the cerebellar cortex; arrow indicates the position where patch clamp pipette is connected to the cell membrane of Purkinje cell; ML – molecular layer, GL – granular layer, lower figure – original trace). UGN effects were not present in GC-C KO animals (**B,** brain slices from wild-type [WT] and GC-C knockout animals, n = 3 each). The hyperpolarization effects of UGN positively correlated with the membrane potential (**B**) – lower panel). UGN did not change the intracellular Ca^2+^concentration of Purkinje cells (**C**), n = 5, from 3 brain slices of 3 animals). Increased K^+^ concentrations were used as a positive control, and increase in Ca^2+^ concentrations due to hyperkalemia (**C**), upper panel) corresponded with the occurrence of action potentials (**C**), lower panel, original trace). Bar represents 10 s. The results are shown as mean ± standard deviation (SD). Asterisk indicates *P* < 0.05 compared with WT mice. ΔF/F_0_ is the change of light output in time (ΔF) over initial brightness (F_0_).

Natriuretic peptides modulate the function of cerebellar Purkinje cells (34), and the cell membrane of these cells expresses GC-C, so we investigated potential roles of GC-C and Ca^2+^ signaling pathway in the cerebellum function. Purkinje cells expressed GC-C (Figure 2B), so it is not surprising that these cells were not hyperpolarized by UGN (100 nM) in GC-C KO animals (WT: -7.2 ± 3.1 mV, n = 5; GC-C KO: 1.6 ± 3.4 mV, n = 4, *t* test: t (7) = 4.074, *P* = 0.0047) (Figure 3). The starting potential of Purkinje cells did not differ between WT and GC-C KO mice (WT: -39.2 ± 8.1 mV, n = 17; GC-C KO: -39.9 ± 5.7 mV, n = 10, *t* test: t (25) = 0.2065, *P* = 0.8381). UGN effects were positively correlated (r = 0.83) to starting membrane potentials (Figure 3). Due to hyperpolarizing effects, UGN significantly decreased the rate of action potentials in Purkinje cells (control: 15.8 ± 2.6 Hz; UGN: 11.6 ± 4.9 Hz, n = 8, t (14) = 2.224, *P* = 0.0431). Since GC-C activation does not involve changes in the intracellular Ca^2+^ concentrations, UGN did not increase the intracellular Ca^2+^ concentration of Purkinje cells. Increased K^+^ concentrations were used as positive control, and the increase in Ca^2+^ concentrations due to hyperkalemia corresponded to the occurrence of action potentials, as previously shown (35) (Figure 3).

**Figure 4:**
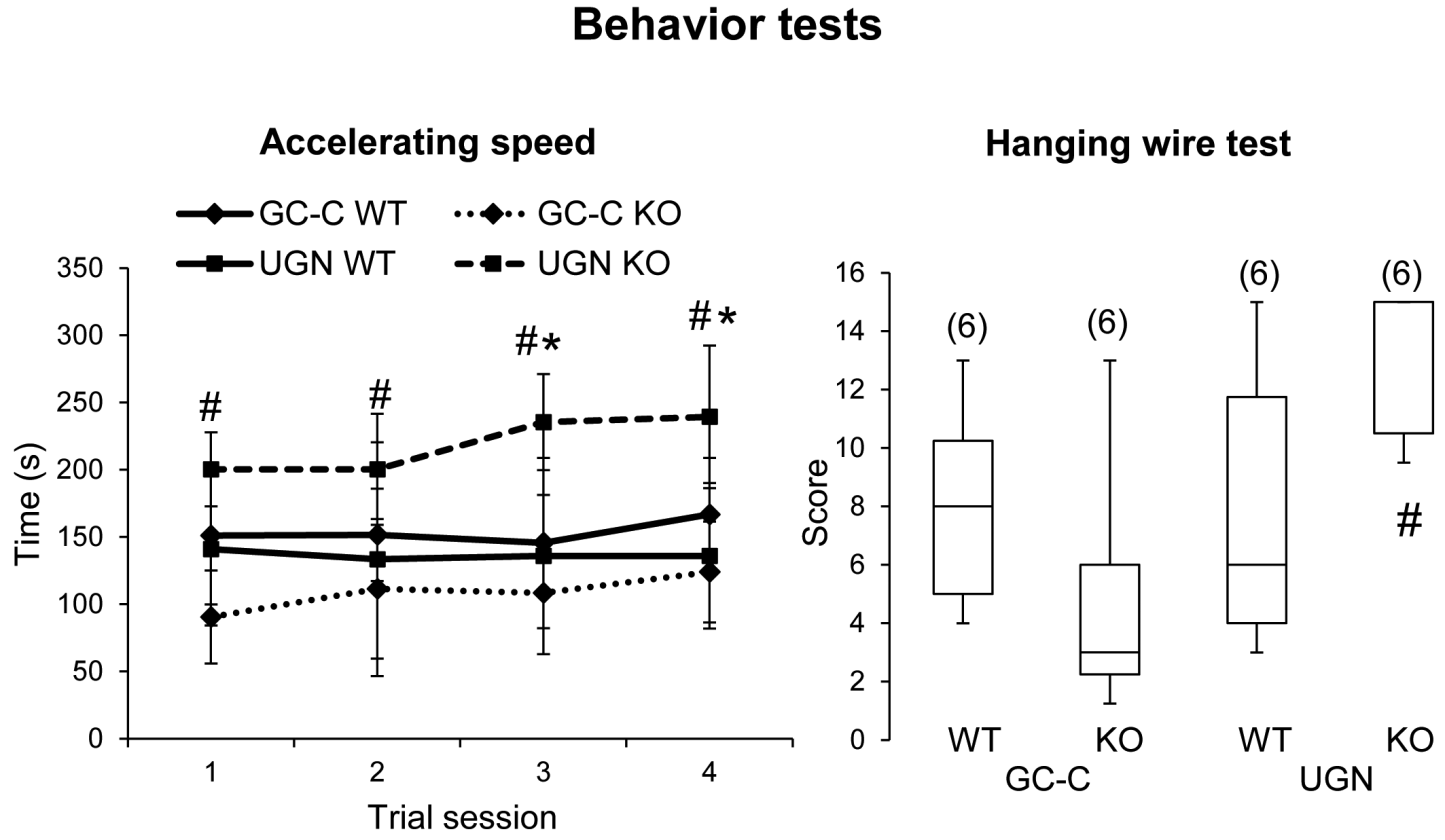
Uroguanylin (UGN) knockout (KO) mice performed better on behavior tests compared with their UGN WT littermates and guanylate cyclase C (GC-C) KO mice. UGN KO mice stayed on the accelerating-speed rota-rod longer than GC-C KO animals. In repeated trials, UGN KO performed even better than UGN WT littermates (panel on the left). The results are shown as mean ± standard deviation (SD). Asterisk indicates *P* < 0.05 compared with WT littermates. On the hanging wire test, GC-C KO mice scored significantly fewer points than UGN KO mice (panel on the right). The results are shown as boxplots displaying the median, quartiles, and extremes. Octothorpe indicates *P* < 0.05 compared with GC-C KO mice (ANOVA *post-hoc *Tukey test). The number of experiments is shown in parentheses.

As shown above, in the cerebellum there exist two signaling pathways for UGN, a GC-C dependent pathway on Purkinje cells and a Ca^2+^-dependent pathway in other cell types, such as astrocytes. To determine the effects of both signaling pathways on balance and strength, we performed behavior tests on GC-C KO and UGN KO animals. These animals differ in the sense that in GC-C KO mice UGN activates Ca^2+^ signaling pathway, while in UGN KO mice, both signaling pathways for UGN are inactivated. GC-C KO animals stayed on the rota-rod shorter that UGN KO mice (GC-C WT: 151 ± 51 s; GC-C KO: 91 ± 34 s; UGN WT: 141 ± 56 s; UGN KO 200 ± 27 s, n = 6, ANOVA: F(3,20) = 6.23059, *P* = 0.0037), reaching the maximum speed of 15.2 ± 3.9 rpm, which was significantly lower compared with UGN KO animals (27.8 ± 3.4 rpm, *t* test: t (10) = 6.001, *P* = 0.0001) (Figure 4). On a repeated test, GC-C KO did not differ from GC-C WT littermates, but in all trials, they performed significantly worse than UGN KO mice. In the third and fourth trial, UGN KO mice performed significantly better than their WT littermates (UGN WT) (second trial: GC-C WT: 151 ± 34 s; GC-C KO: 112 ± 52 s; UGN WT: 133 ± 87 s; UGN KO 200 ± 41 s, n = 6, ANOVA: F(3,20) = 2.61318, *P* = 0.07953; third trial: GC-C WT: 146 ± 36 s; GC-C KO: 108 ± 26 s; UGN WT: 136 ± 73 s; UGN KO 236 ± 36 s, n = 6, ANOVA: F(3,20) = 8.50209, *P* = 0.000771; fourth trial: GC-C WT: 167 ± 42 s; GC-C KO: 124 ± 37 s; UGN WT: 136 ± 54 s; UGN KO 239 ± 53 s, n = 6, ANOVA: F(3,20) = 7.21935, *P* = 0.00181). Similar results were observed on the hanging wire test, where GC-C KO mice scored significantly fewer points than UGN KO mice, in which 4 out of 6 scored the maximum 15 points (GC-C WT: median [IQR] = 8 [5.25]; GC-C KO: median [IQR] = 3 [3.75]; UGN WT: median [IQR] = 6 [7.75]; UGN KO: median [IQR] = 15 [4.5], n = 6, Kruskal-Walls test with *post-hoc* Dunn test, *P* = 0.0182).

### Uroguanylin activates GC-C-independent but Ca^2+^-dependent signaling pathway in astrocytes

**Figure 5:**
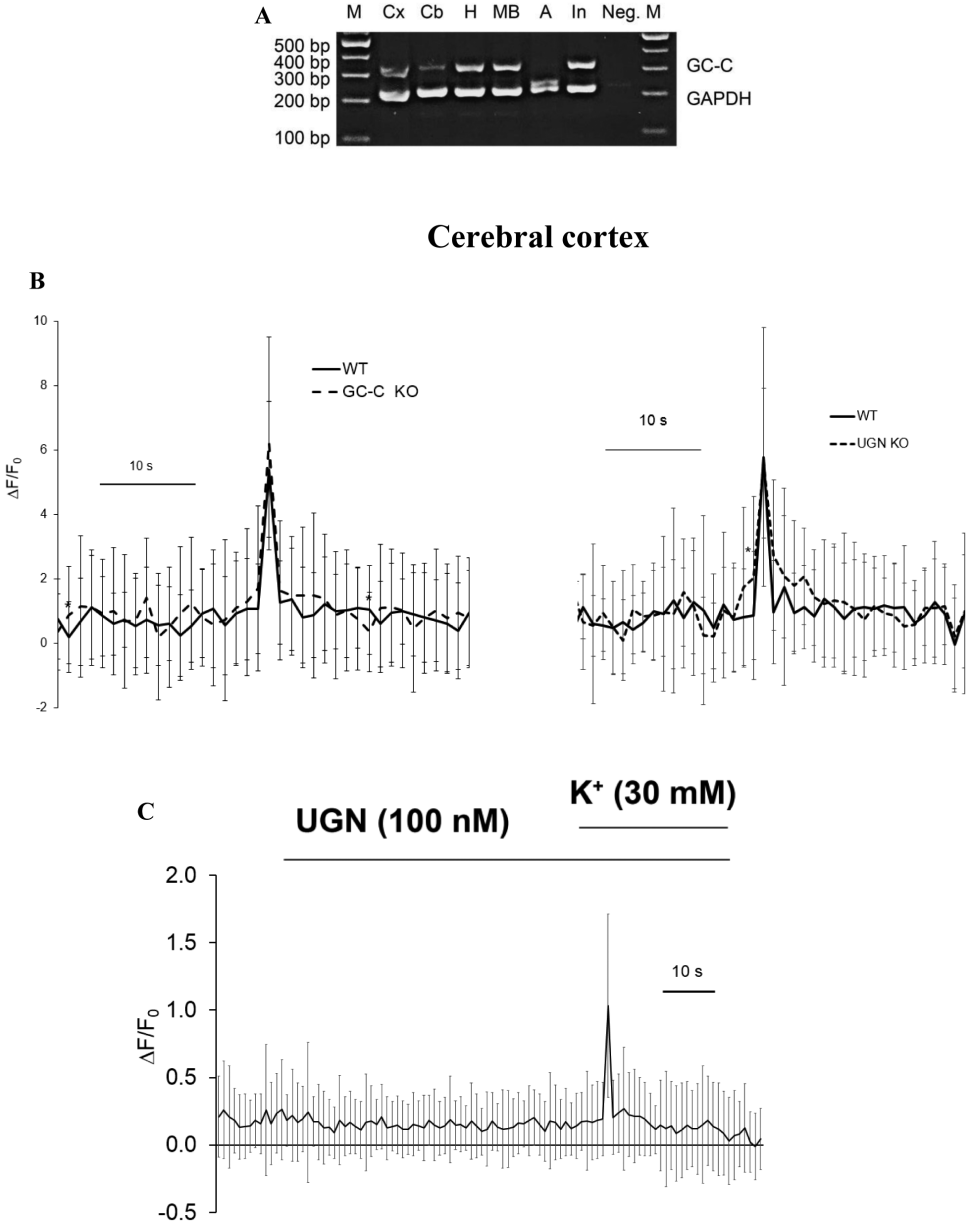
Uroguanylin (UGN) Ca^2+^ signaling pathway in the cortical astrocytes of brain slices. mRNA of guanylate cyclase C (GC-C) (341 bp) is expressed in the hypothalamus (H), midbrain (MB), cerebral cortex (Cx), and cerebellum (Cb) but not in astrocytes (A) (**A**). M – marker, In – intestine as positive control, Neg – negative control, glyceraldehyde 3-phosphate dehydrogenase (GAPDH, 235 bp) was used as cDNA control. Ca^2+^ measurements were performed only in SR101-positive cells (**B**) of GC-C knockout (KO) (left, the number of animals is 3, the number of brain slices is 6, the number of cells is 32) and UGN KO (right, the number of animals is 3, the number of brain slices is 5, the number of cells is 14) cerebral cortex, and the results are compared with those of their wild-type littermates (GC-C WT: the number of animals is 4, the number of brain slices is 6, the number of cells is 28; UGN WT: the number of animals is 3, the number of brain slices is 5, the number of cells is 17). In neurons of the cerebral cortex of WT animals, UGN did not affect the intracellular Ca^2+^ concentration (the number of animals is 4, the number of brain slices is 8, the number of cells is 43). Hyperkalemia of 30 mM was used as a positive control (**C**). The results are shown as mean ± standard deviation (SD) ΔF/F_0_ is change of light output in time (ΔF) over initial brightness (F_0_). Bar represents 10 s.

Similarly to GC-C protein expression, GC-C mRNA expression was observed in the cerebral cortex (33), cerebellum, hypothalamus (10-13), and midbrain (9) but not in isolated astrocytes (Figure 5A). Since in the cerebral cortex of GC-C KO animals Ca^2+^ response to UGN still exists but lasts significantly shorter (Figure 1B), we examined this response in SR101-positive cells (marker for astrocytes) in the cerebral cortex of GC-C KO and UGN KO mice and their WT littermates. The results are presented as a ratio to starting values. Astrocytes' Ca^2+^ response to UGN in GC-C KO and UGN KO mice did not differ when compared with their WT littermates (Figure 5B). In addition, some SR101-negative cells showed no increase in the intracellular Ca^2+^ concentration upon UGN stimulation (Figure 5C), similarly to the results observed for cerebellar Purkinje cells.

**Figure 6:**
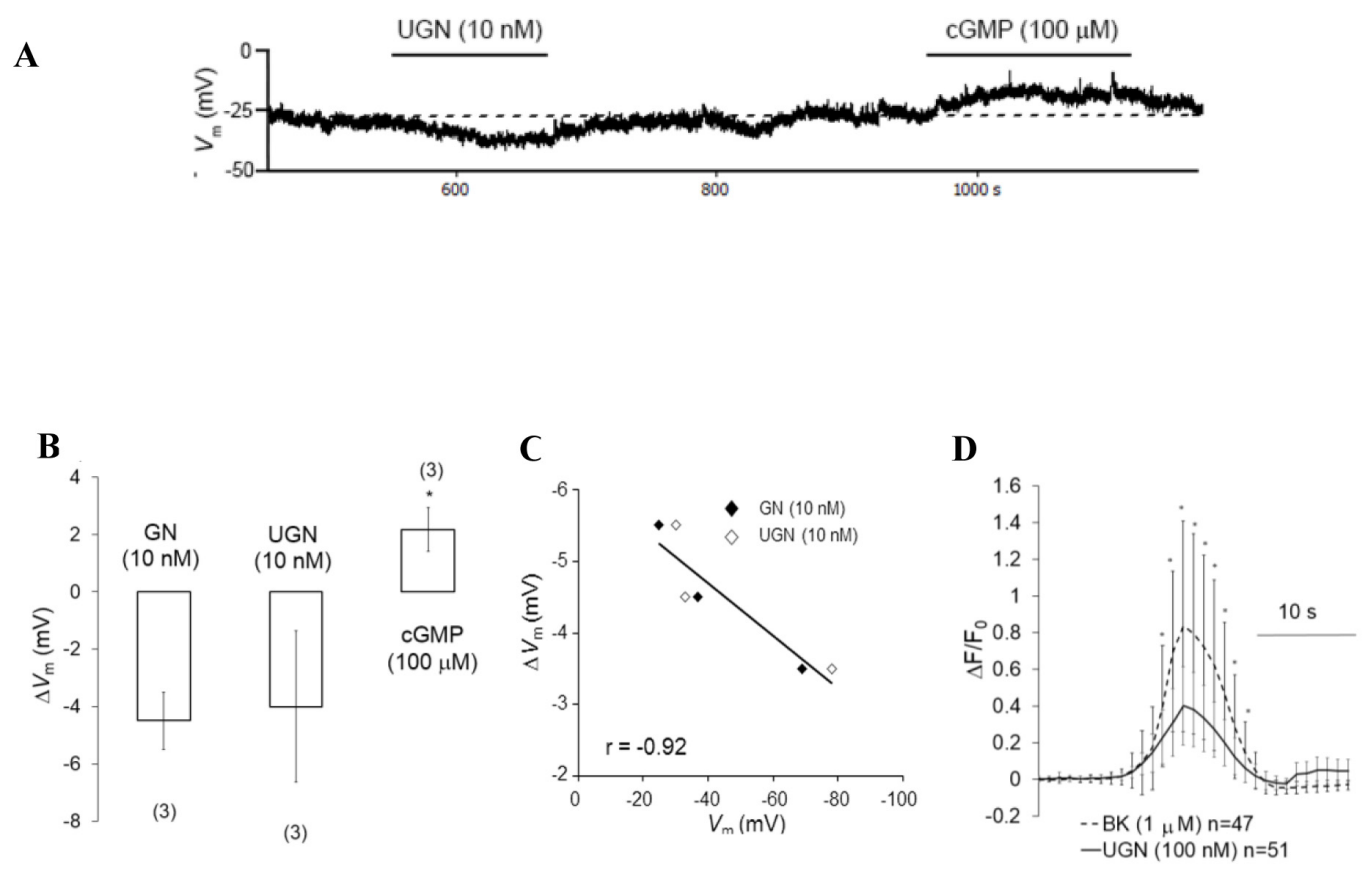
Uroguanylin (UGN) Ca^2+^ signaling pathway in primary astrocyte cultures. An electrophysiological recording showing an effect of UGN (10 nM) opposite to that of membrane permeable cyclic guanosine monophosphate (cGMP, 100 μM) on astrocyte membrane potential – original trace. Dashed line represents starting membrane potential (**A**). Guanylin (GN, 10 nM) and UGN hyperpolarized, while membrane permeable cGMP depolarized astrocytes (**B**). Hyperpolarization caused by guanylin peptides was negatively correlated with the starting membrane potential (**C**). UGN (100 nM) and bradykinin (BK, 1 μM – positive control) increased intracellular Ca^2+^ concentration (**D**). The results are shown as mean ± standard deviation (SD). The number of experiments is shown in parentheses. Octothorpe indicates a significant difference between cGMP effects and effects of guanylin peptides, at *P* < 0.05. Asterisk indicates a significant difference between UGN and BK effects, at *P* < 0.05. ΔF/F_0_ is change of light output in time (ΔF) over initial brightness (F_0_). Bar represents 10 s.

To better characterize the UGN Ca^2+^ signaling pathway, we used the primary astrocyte culture. Astrocytes were hyperpolarized by GN and UGN (10 nM, each) (-4.5 ± 1.0 mV and -4.0 ± 2.7 mV, n = 3, respectively) (Figure 6). These effects were negatively correlated (r = -0.92, *P* = 0.0092) with the starting membrane potential. In the paired experiments, the same cells were depolarized by membrane permeable cGMP (8 Br cGMP, 100 μM) (2.2 ± 0.8 mV, n = 3). Since cGMP is not a second messenger for GPs in astrocytes, to confirm the existence of Ca^2+^ signaling pathway, we performed Ca^2+^ imaging, which showed that UGN (100 nM) increased the intracellular Ca^2+^ concentration in these cells (Figure 6). BK (1 μM), used as positive control, showed significantly weaker effects (32).

### Uroguanylin changes transport of H^+^ and bicarbonate

The possible physiological importance of this novel signaling pathway lies in the regulation of different membrane transporters. The astrocyte Ca^2+^ signaling pathway is involved in the regulation of bicarbonate transporters, which affect the intracellular and extracellular pH (36). Therefore, we examined the effect of UGN on intracellular astrocyte pH (pH_i_). UGN application increased pH_i_ recovery slope after ammonia pulse by 70% due to an increase in NHE activity (*t* test: t (7) = 2.429, *P* = 0.0455) (Figure 7). Furthermore, UGN increased the cell alkalization slope 2.5-fold after astrocyte exposure to CO_2_/HCO_3_^-^ (*t* test: t(5) = 2.711, *P* = 0.0422), suggesting that UGN activates HCO_3_^-^ transport (Figure 7).

## Discussion

This study for the first time showed the presence of two signaling pathways in the cerebellum, the already known GC-C signaling pathway and a GC-C-independent but Ca^2+^-dependent signaling pathway. The existence of these two pathways led to a different motoric function in GC-C KO and UGN KO mice, probably via different regulation of intracellular pH in astrocytes.

Besides GC receptor, there exists another receptor for natriuretic peptides. The activation of this additional signaling pathway increases the intracellular Ca^2+^ and cAMP concentrations (37). In the cell culture of human proximal kidney cells, GC-C-independent signaling pathway for GPs involves the activation of pertussis toxin-sensitive G protein-coupled receptor (19). The physiological importance of this cGMP-independent but Ca^2+^-dependent signaling pathway for GPs and its existence in the brain has not been investigated so far.

Since GC-C was found in the cerebral cortex (33), cerebellum, hypothalamus (10-13), and midbrain (9), we performed Ca^2+^ imaging experiments on brain slices of WT and GC-C KO mice in different brain regions. In the cerebral cortex, but not in the cerebellar cortex, UGN effects lasted significantly shorter in GC-C KO mice than in WT animals. The GC-C effects on Ca^2+^ signaling could be explained by the ability of some brain cells (like neurons) to express both signaling pathways for UGN. The difference in Ca^2+^ response to UGN between GC-C KO and WT mice was abolished in the SR101-positive cells (astrocytes) of the cerebral cortex, supporting the hypothesis that both signaling pathways could exist in the same cells. In the cerebellar cortex, UGN did not change the intracellular Ca^2+^ concentrations of Purkinje cells. It is not surprising that UGN hyperpolarized Purkinje cells, which express GC-C, in WT but not in GC-C KO animals. Hyperpolarization decreased the rate of action potentials. However, even though GC-C KO mice show attention deficiency and hyperactive behavior (9), they did not perform significantly differently on behavior tests. This finding is not surprising since a previous study found no differences in rota-rod performance between rats with ADHD and healthy animals (38). Therefore, hyperactivity cannot explain poor performance. On the other hand, UGN KO mice, in which both signaling pathways did not activate, performed much better than GC-C KO and UGN WT littermates. This corresponds to previous findings that one of the physiological functions of Ca^2+^ signaling pathway in astrocytes is the regulation of motor function (22). Furthermore, to better understand the negative effects of UGN via Ca^2+^ signaling pathway, we studied the primary astrocyte culture. In astrocytes, UGN decreased the extracellular pH via increasing the NHE activity and via bicarbonate removal from the extracellular fluid. Therefore, we can hypothesize that extracellular alkalinization due to lack of UGN effects on astrocytes in UGN KO mice increases neuronal activity, which could explain their better performance on behavioral tests.

Since GC-C is not expressed in astrocytes, the membrane potential was changed by GPs, demonstrating the existence of a GC-C/cGMP-independent signaling pathway. GPs could hyperpolarize astrocytes via an increase in Cl^-^ or K^+^ conductance since the effects of GPs were negatively correlated with the starting membrane potential. We showed that UGN increased the intracellular Ca^2+^ concentration, which could activate Ca*^2+^*-dependent Cl^-^ channels or Ca*^2+^*-regulated K^+^ channels (39-41). In contrast to the effects of GPs, membrane permeable cGMP depolarized the same cells probably by inhibiting K^+^ conductance, as previously shown (19-21,42).

Changes in H^+^ and HCO_3_^-^ transport affect pH_i_ and the pH of brain extracellular fluid (pH_e_). Since H^+^ inhibits N-methyl-D-aspartate glutamate receptors and voltage-gated Ca^2+^ channels, pH_e_ alkalinization or acidification can increase or decrease neuronal activity (43,44). cGMP is known to decrease astrocyte pH_i_ by NHE inhibition (45). In other cell models, UGN inhibits H^+^ transport (NHE, H^+^-ATP-ase) via cGMP (46,47) and changes the expression of Cl^-^/HCO_3_^-^ exchanger (Slc26a4) (48). Since we established that cGMP was not a second messenger for UGN in astrocytes, H^+^ and HCO_3_^-^ transport via Ca^2+^-signaling pathway could be regulated differently (49). Indeed, UGN application increased the pH_i_ recovery slope after the ammonia pulse by increasing the NHE activity and increased the cell alkalization slope after the exposure of astrocytes to CO_2_/HCO_3_^-^, suggesting the activation of HCO_3_^-^ transport. NHE and HCO_3_^-^ transporters are involved in the pathophysiology of ischemic injury; however, further research is needed to define the possible involvement of UGN in the development of brain ischemic injury (50-52).

The presented results suggest a neurophysiological importance of GC-C-independent signaling pathway for UGN in astrocytes. Our study suggests that UGN has various effects on neurons since it changes the membrane potential and decreases the action potential rate of cerebellar Purkinje cells. In addition to GC-C-dependent signaling pathway in neurons, in astrocytes, UGN binds to a GC-C-independent receptor, whose activation increases the intracellular Ca^2+^ concentration. The presence of two signaling pathways in the cerebellum was additionally proved by a better motoric function of UGN KO compared with GC-C KO mice. UGN, via a novel GC-C-independent signaling pathway, in astrocytes regulates the intracellular and extracellular pH by increased NHE activity and HCO_3_^-^ transport.
